# Function of second cladding layer in hollow core tube lattice fibers

**DOI:** 10.1038/s41598-017-01839-5

**Published:** 2017-05-09

**Authors:** Xiaosheng Huang, Seongwoo Yoo, KenTye Yong

**Affiliations:** The Photonics Institute, School of Electrical and Electronics Engineering, Nanyang Technological University, Singapore, 639798 Singapore

## Abstract

Modes attenuation of the tube lattice fiber (TLF) is characterized by *D*/λ, where *D* is the core diameter and *λ* is the wavelength. Hence, the TLF is structured with a large core to ensure a low attenuation loss. A small core, on the other hand, facilitates the gas-filled TLF applications, but at the expense of the increased mode attenuation. We show that adding a second cladding layer to the conventional one layer TLF (1TLF) can resolve the contradicting requirements. The mode attenuation of TLF with two cladding layers (2TLF) is less influenced by the *D*/*λ* value as compared to 1TLF, thus realizing a low loss small core TLF. Furthermore, we found that adding the second layer brings another advantage to a bending performance. With a determined core size, *D*, a 1TLF with smaller capillary hole size, *d*, experiences less bending loss. However, the reduced *d* increases the confinement loss that counteracts the bending loss improvement. This confliction is substantially alleviated in 2TLF thanks to the second cladding layer. Theoretical investigations and experimental demonstrations are presented to evidence the important role of the second cladding ring in the TLF, which has been overlooked in prior studies.

## Introduction

Hollow core photonic crystal fibers (HC-PCFs) have attracted a significant interest because of their novel characteristics that offer new ways to deliver light^1–5^, sense chemicals^6, 7^, exploit gas-based nonlinear optics^8–11^, and generate suppercontinuum^12^, to name a few. Light guidance via the air core in the HC-PCFs is typically achieved by utilizing a two dimensional photonic bandgap^13^ or antiresonant reflection (or inhibited coupling)^14–16^. In particular, the first antiresonant fiber was experimentally demonstrated as a Kagome Fiber (KF)^17^ which was constituted from a multiple layers of air holes running down the fiber. It was proven that optical properties of the KF largely depend on the first air cladding layer surrounding the air core^18^. Subsequently, a simplified antiresonant structure with one air cladding layer has been widely investigated as a hollow core antiresonant fiber^19, 20^ or an inhibited coupling fiber^21^ where the inhibited coupling model offers more precise description of the fiber guidance^15, 16^. The simplified structure features a negative core curvature, a non-touching core boundary and one tube cladding layer^16, 19, 22, 23^, thus often being named as Tube Lattice Fiber (TLF)^24–26^. The negative curvature is found to enhance the coupling inhibition between the core and cladding modes^27–29^ while the non-touching core boundary helps to reduce a loss caused by the Fano-resonance^21, 30^. Moreover, the simple one ring cladding is proven sufficient to offer efficient light guidance^18, 31^. Consequently, the first ring has been the emphasis of a TLF design. Nonetheless, the role of a second cladding layer on confinement loss reduction cannot be thoroughly ignored^32, 33^.

Recently, a record transmission loss of 7.7 *dB*/*km* at 750 *nm* in a TLF has been demonstrated^16^, but the transmission loss was compromised as a wavelength increases, e.g. 70 *dB*/*km* at 1500 *nm*. This is attributed to the characteristics of the TLF where a confinement loss (CL) is associated to the value of *D*/*λ*, where *D* is a core diameter, and *λ* is a transmission wavelength. As a transmission window moves to longer wavelength or a core size gets reduced, the CL instantly increases^34, 35^. Hence, a large core is a typical feature in the TLF. Although the large core certainly helps to reduce the CL, it can sometimes contradict to other requirements. For instance, a small core can increase pump light intensity and ameliorate high power requirement to overcome a threshold in gas-filled TLF applications. Thus, a small core TLF without compromising the CL could be a next important step to advance the gas-filled TLF technology.

This work highlights an alternative design to mitigate this contradiction. We present a two layer TLF (2TLF) with an emphasis on the important role of the second cladding layer. Unlike a one layer TLF (1TLF), the 2TLF benefits from the presence of the second layer to suppress the CL even in a small core structure. We theoretically and experimentally compare a transmission loss of the 1TLF and the 2TLF over several transmission bands to confirm the critical role of the second ring. Moreover, the second ring is also found to improve a bending loss. A small capillary hole size is known to suppress the bending loss^19, 36^, but the small hole also increases the CL, hence compensating the already improved bending loss from the small hole. We present benefits of the 2TLF to resolve this contradiction. The dependence of the bending loss on the hole size is also experimentally demonstrated. Consequently, 2TLF is found to fulfill the contradicting requirements of a small core, low bending loss and low transmission loss. This paper contributes to an alternative design route, with a potential of gas-filled TLF applications and easier fiber handling.

## Results

### Suppression of CL in a 2TLF

**Figure 1:**
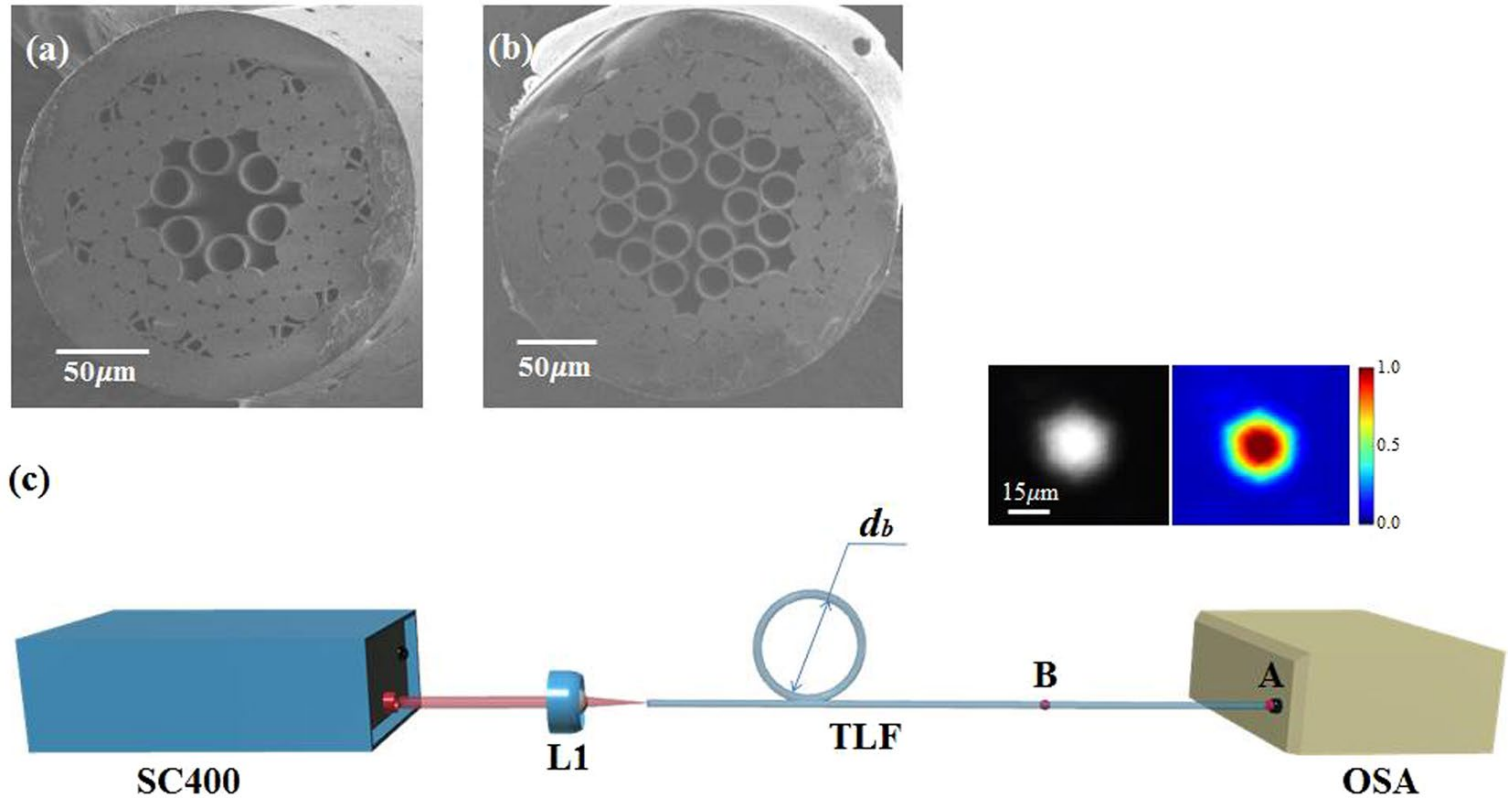
Microscopic images of the cross-section of (**a**) 1TLF, with 32.4 *μm* core diameter, 1.45 *μm* wall thickness, 20.3 *μm* capillary hole diameter and 220 *μm* outer diameter, denoted as Fiber #1; (**b**) 2TLF, with 31.9 *μm* core diameter, 1.96 *μm* wall thickness, 20.9 *μm* capillary hole diameter and 245 *μm* outer diameter, denoted as Fiber #2; (**c**) Schematic of the setup used to measure the transmission loss of the fundamental mode, based on the cut-back method. The fiber is first bent to a smaller circle with *d*_*b*_ = 15 *cm*, in order to filter out the higher-order modes. *P*_*A*_ is the power at point A, *P*_*B*_ is the power at point B, *l* is the cut fiber length between point A and B. The fiber loss is then calculated as $\frac{{P}_{B}-{P}_{A}}{l}$. The inset shows the near field mode image at point B, a pure fundamental mode can be observed. SC400: supercontinuum laser source, L1: microscope objective, TLF: tube lattice fiber, OSA: optical spectrum analyzer.

Two tube lattice fibers, a 1TLF and a 2TLF, are compared to demonstrate the role of the second cladding layer. We used a stack-and-draw method to fabricate the fibers. The structure uniformity was maintained by building precise differential pressure during a fiber drawing process. Figuire 1 represents the fabricated fibers. A 1TLF (Fiber #1) is measured as 32.4 *μm* core diameter, 20.3 *μm* capillary hole size and 1.45 *μm* wall thickness. A 2TLF (Fiber #2) has 31.9 *μm* core diameter, 20.9 *μm* capillary hole size and 1.96 *μm* wall thickness as shown in Fig. 1(b). Both fibers are in good uniform structure within a 5 % of variation in core diameters. The two fibers are made into similar core size on purpose for a fair loss comparison. The conventional cutback method^37^ is applied to measure the background loss of the fundamental mode (FM) of the fibers. As is shown in Fig. 1(c), the fiber is bent to a circle with 15 *cm* diameter in order to filter out the higher-order modes (HOMs). One end facet of the TLFs is used for a signal input end, and the other facet is served as a signal output end. A near field mode image is monitored from the output facet with a CCD camera to monitor the fundamental mode propagation (Please, see the inset in Fig. 1(c)). Thus, the loss measurement only includes a robust FM. Subsequently, the FM output power spectrum is collected by an optical spectrum analyzer (OSA).

**Figure 2:**
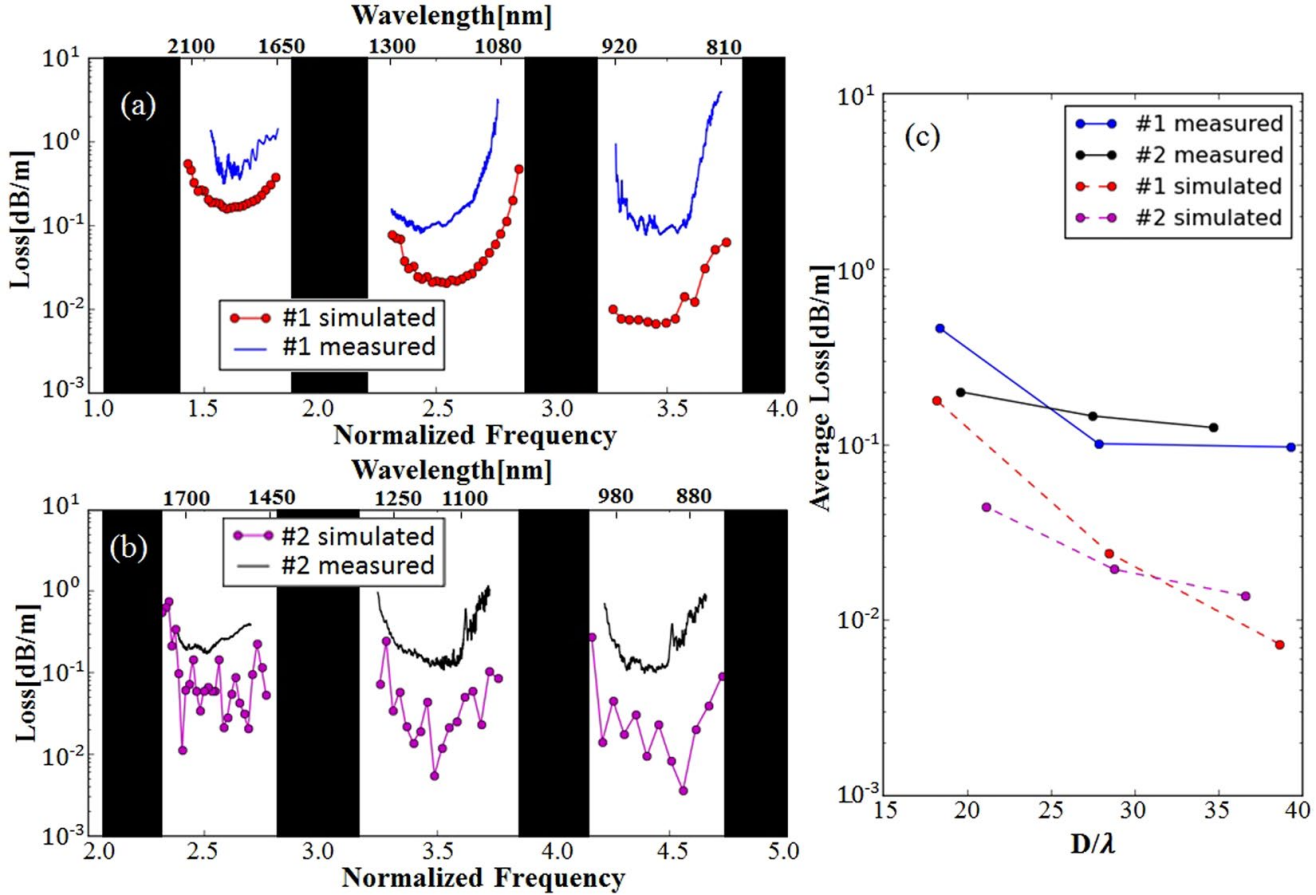
(**a**) Simulated and measured loss evolution of Fiber #1 over three transmission bands (800 *nm* to 2100 *nm*), (**b**) simulated and measured loss curves of Fiber #2 over three transmission bands (850 *nm* to 1750 *nm*), black areas represent the high resonant regions; (**c**) average loss taken at different transmission bands over *D*/*λ* value. In all the figures, blue and red lines show the measured and simulated loss of Fiber #1 respectively, while black and magenta lines show the measured and simulated loss of Fiber #2 respectively. Here, simulated loss represents the CL only while the measured loss represents the total fiber loss including the CL and the fabrication induced loss.

The loss of the Fiber #1 (1TLF) measured with 20 and 3 *m* fibers is shown by the blue curve in Fig. 2(a). The loss of the Fiber #2 measured with 15 and 3 *m* fibers is shown by the black curve in Fig. 2(b). The dotted curves in Fig. 2(a) and (b) represent simulated CL obtained by a vector wave expansion method using the open source software Polymode^38, 39^. Our simulation code can reproduce CL of a TLF reported in 2014^40^ within 15 % discrepancy. The losses are plotted over both the normalized frequency, $F=2t\sqrt{{n}^{2}-1}/\lambda$, and wavelength, *λ*, where *t* is the core wall thickness, *n* is the refractive index of cladding material. When *F* closes to an integer, there is a high loss region, and a low loss transmission band exists between every adjacent high loss regions^15, 26^. The CL is determined by a fiber design, but there are other contributors to the loss introduced during fabrication such as a scattering loss^13, 34, 40^ and an imperfection loss induced by structural variation across the fiber cross-section such as uniformity of capillary holes or hole distance^34^. We note that the fabrication induced losses reduce with increasing wavelength (thus, decreasing *D*/*λ*)^34, 40^. Consequently, it is the CL that contributes to the increasing measured loss when the value of *D*/*λ* gets smaller toward longer wavelength. At a short wavelength region, the fabrication related losses contribute more to the evolution of the total fiber loss than the CL^34^. This explains the insignificant loss difference between 1TLF and 2TLF at the short wavelength band in Fig. 2. When transmission band moves towards longer wavelength, the CL grows quickly and becomes a dominant factor to the total fiber loss. Such tendency is evident in 1TLF (Fig. 2(a)), but not in 2TLF (Fig. 2(b)) for both simulation and experimental results.

The behaviors of the measured loss and the simulated CL over *D*/*λ* are summarized in Fig. 2(c). The presented loss at each band was obtained by taking an average of the loss curves in Fig. 2(a) and (b) excluding the sharp rises close to the inhibited regions. As demonstrated in Fig. 2(c), the second cladding layer slows the loss increasing rate over the *D*/*λ*, and making the 2TLF as a promising design to applications benefiting from a small *D*/*λ*, or a small core. Apparently, the loss with a smaller *D*/*λ*, or in a longer wavelength, is lower in 2TLF. A quality of TLF for gas-light interaction applications can be determined by introducing a figure of merit (FOM) i.e., ${f}_{om}=\frac{\lambda }{\pi {D}^{2}\alpha }$, where *α* is the exponential attenuation of a propagation light intensity^17^. From the measurement results in Fig. 2(c), the FOM of both Fiber #1 and Fiber #2 is around 2500 when *D*/*λ* = 27. However, as the value of *D*/*λ* decreases to 20, the FOM of Fiber #2 maintains the same, whereas that of the Fiber #1 decreases to 1240. Thanks to the lower attenuation in the long wavelength band as well as the small core, the 2TLF could offer better performance in the gas-light interaction applications.

### Demonstration of relationship between capillary hole size and bending loss

A prior theoretical study^19^ reported that a bending loss of 1TLF is greatly influenced by cladding hole size in that a small hole size suppresses the bending loss at a given core size. The smaller tube hole size reduces an effective refractive index of a cladding airy mode^24^, rendering coupling between core and cladding modes weaker^20, 25^, even in a bent fiber. We provide experimental evidences of this theoretical investigation. Bending losses in 2TLFs with various hole sizes are experimentally examined to confirm the relationship.

**Figure 3:**
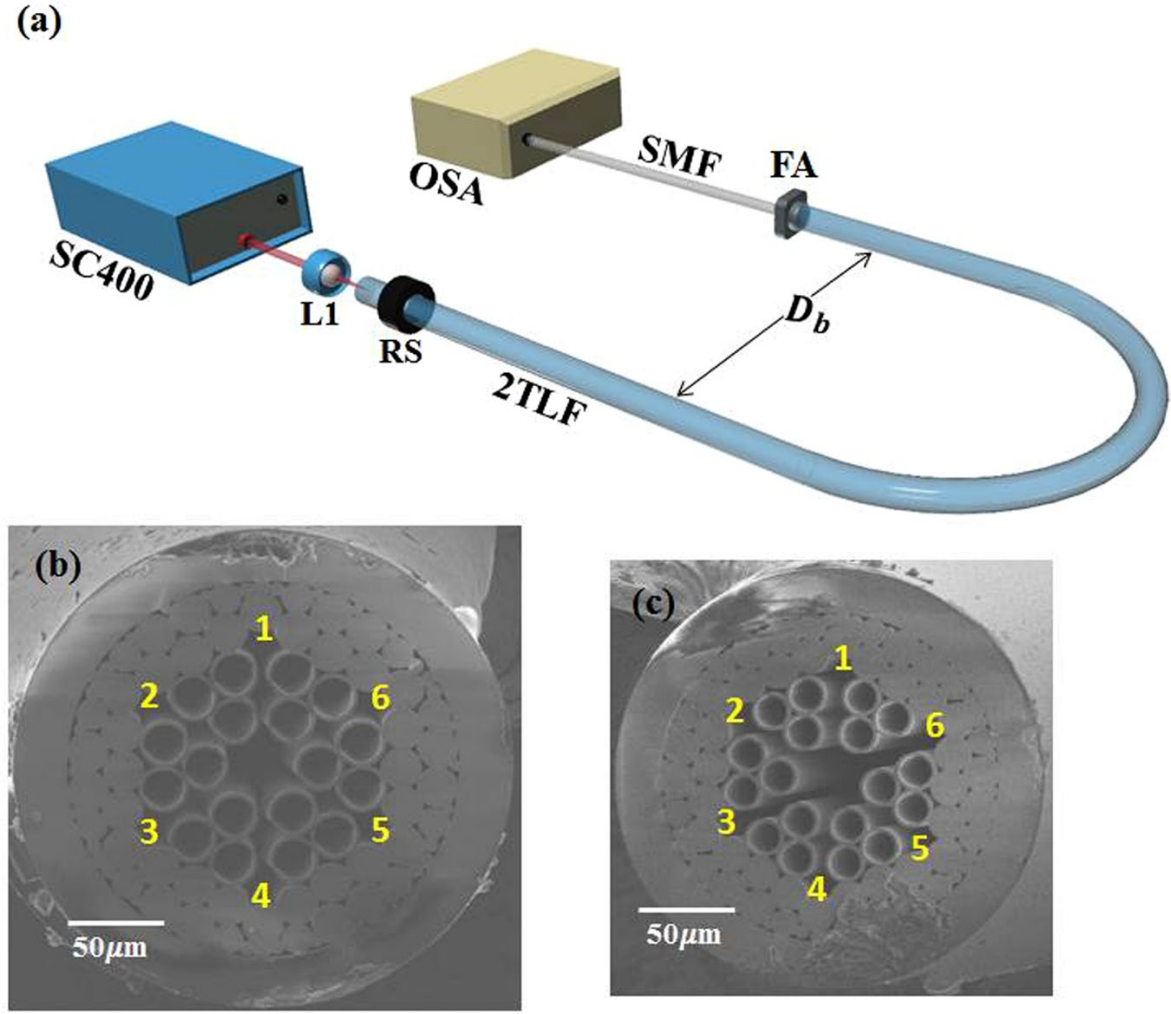
(**a**) Schematic of the setup used to measure the bending loss, SC400: supercontinnuum laser source, L1: microscope objective, RS: rotational stage, FA: fiber adapter, SMF: single mode fiber, OSA: optical spectrum analyser; Cross-section image of (**b**) Fiber #3 with core diameter *D* = 32.0 *μm*, capillary hole diameter *d* = 19.7 *μm*, $\frac{d}{D}=0.62$; (**c**) Fiber #4, core diameter *D* = 32.3 *μm*, capillary hole diameter *d* = 16.0 *μm*, $\frac{d}{D}=0.50$. Gaps in the cladding area are denoted with numbers in a yellow font.

The schematic of our bending loss measurement is depicted in Fig. 3(a). A fiber laser pumped supercontinuum source is coupled into the 2TLF which is bent to a half circle with diameter *D*_*b*_. After the bending, only a core mode is guided. The core mode is coupled into a telecomm single mode fiber (SMF) and a transmission spectrum is recorded by an OSA. Because the bending loss can be subject to the bending direction in our 2TLF, 2TLFs under test are mounted on a rotational stage in order to align the bending direction to the split gap. The fiber #3 and #4 in Fig. 3(b) and (c) have a similar core size, *D*, around 32 *μm*, but different capillary hole diameters, *d*, of 19.7 and 16.0 *μm*, respectively. Thus, their *D*/*d* parameter is determined by *d* only. The wall thickness of both fibers is same at 2.0 *μm*. The two fibers are experimentally characterized with a similar background loss around 0.2 *dB*/*km* at 1200 *nm* because of the similarity in the core size and the wall thickness.

**Figure 4:**
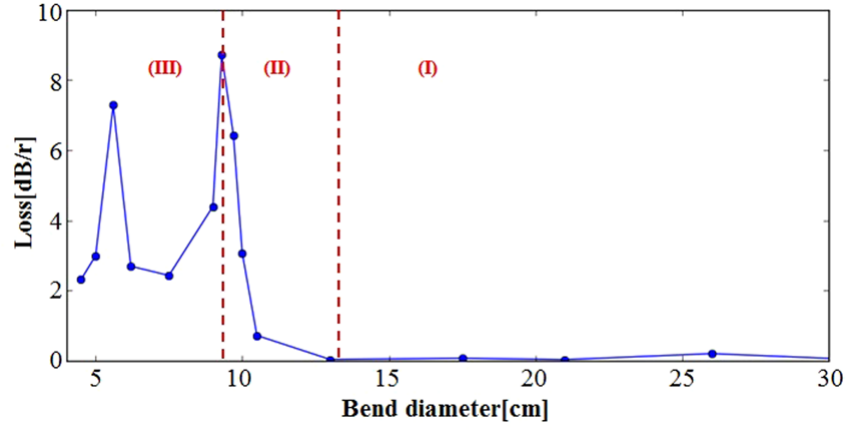
Shows the bending loss of Fiber #3 at different bending diameters at 1200 *nm*. Region **I**: insignificant bending loss, region **II**: bending loss increases exponentially, region **III**: bending loss oscillates.

The evolution trend in bending loss of Fiber #3 is shown in Fig. 4 when the fiber bending direction is aligned to a split gap. The bending loss curve is divided into three parts according to the evolution trend. When the bending diameter is large (region **I**), the bending loss is too low to be accurately measured. However, prior simulation works reported the bending loss at this region increases exponentially^19^. When the bending diameter enters region **II**, its bending loss grows enough to be detected by the OSA. An exponential increase of the bending loss with tighter bending is apparent in this region as shown in Fig. 4. As we further reduce the bending diameter (region **III**), the behavior dramatically changes. Instead of the exponential growth, the bending loss oscillates in an irregular manner. This behavior was already predicted in simulation with an explanation of the bending dependence of the Fano resonances^21, 33^. Our experimental observation confirms the theoretical results. We also note that a similar behavior was observed regardless of the bending direction to different split gaps. As our main interest is to attain experimental evidences of the bending loss dependence on the hole size, the experiments were carried out in the Region **I** and **II** only. To evaluate the bending loss, we introduce a 3 *dB* diameter, i.e., a bending diameter at which a bending loss becomes 3 *dB*/*round*. Thus, the smaller the 3 *dB* diameter, the lower the bending loss.

**Figure 5:**
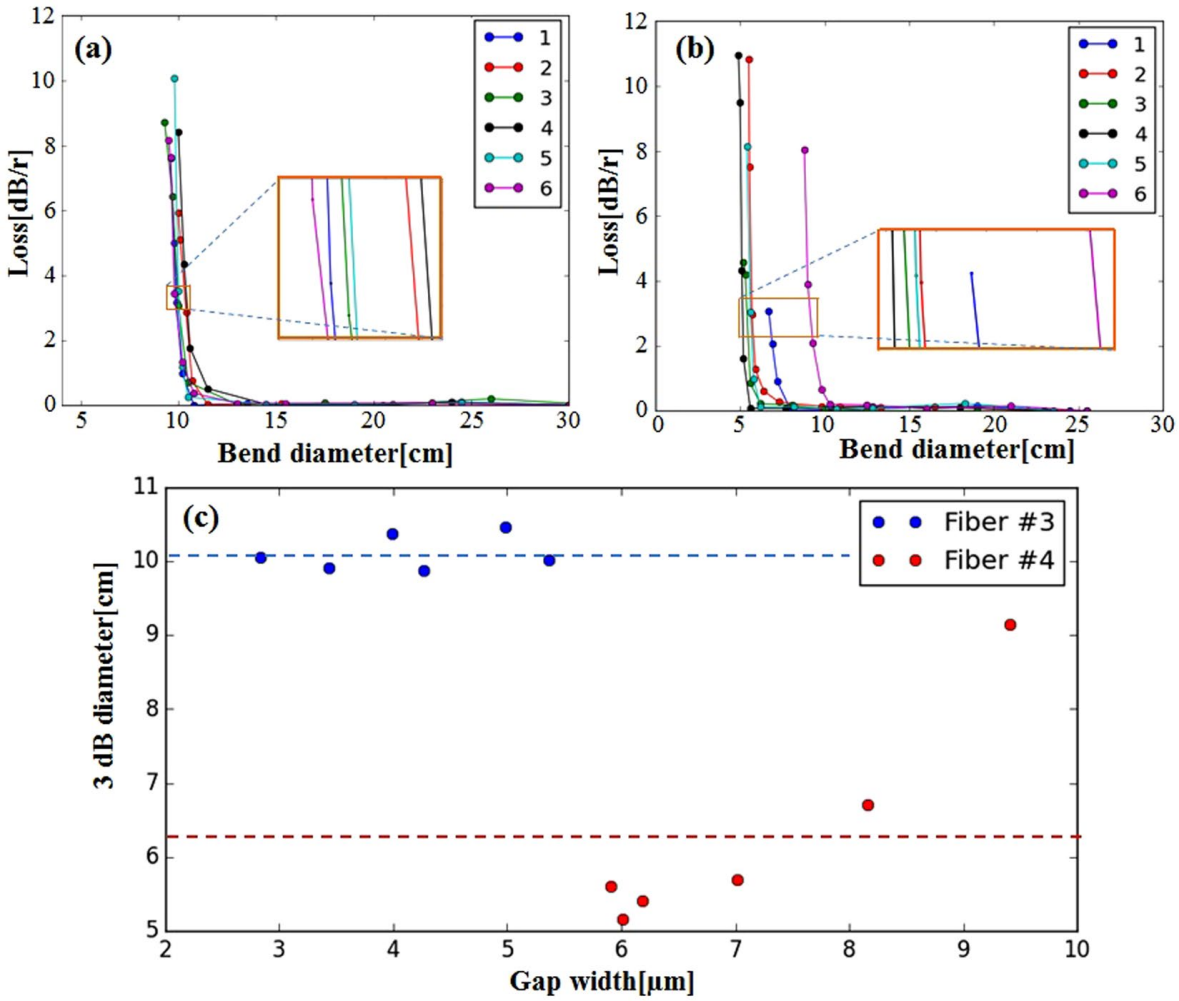
Bending loss of (**a**) Fiber #3 and (**b**) Fiber #4 at 1200 *nm* (F = 3.5) when bent to different directions; (**c**) The relationship between 3 dB diameter and gap width (or azimuthal separation of cladding). Blue colors correspond to the Fiber #3 $(\frac{d}{D}=0.62)$ while red colors correspond to the Fiber #4 $(\frac{d}{D}=0.50)$. Dash lines are the average 3 dB diameter values.

Figure 5(a) and (b) represent measured bending losses of Fiber #3 and Fiber #4 respectively at different bending directions. The tested fibers were bent to different split gaps as labeled from 1 to 6 in anti-clockwise direction in Fig. 3. The bending performances are then summarized in Fig. 5(c). The average 3 *dB* bending diameter of Fiber #3 with *d*/*D* = 0.62 is measured as 10.1 *cm*, which is 60 % larger than Fiber #4 (*d*/*D* = 0.50) whose 3 *dB* bending diameter is averaged to 6.3 *cm*. Since the two fibers have similar core size (around 32.0 *μm*) and consequently the same loss around 0.2 *dB*/*m* at 1200 *nm*, the only variable in *d*/*D* becomes the hole size, *d*. We note that the hole size of Fiber #3 and Fiber #4 are measured as 19.7 and 16.0 *μm* respectively. Therefore, the result clearly demonstrates that the fiber with a smaller hole diameter indeed enjoys lower bending loss.

We now clarify the bending loss dependence on the bending directions in Fig. 5(a) and (b). Compared to Fiber #3 (Fig. 5(a)), the bending direction dependence is more significant in Fiber #4 (Fig. 5(b)). This is caused by asymmetric nature of fiber structure. While the split gaps are only varied within 2.0 *μm* in Fiber #3, the gap size in Fiber #4 exhibits wider variation of 4.0 *μm*. As a consequence, the bending loss in Fiber #4 becomes subjective to the bending directions. Bending to a wider gap suffers larger bending loss as found in Fig. 5(c) (red dots). Our observation suggests the wider gap can bridge the mode index difference between the core and the cladding modes and increases the bending loss^20^.

### Low bending loss and low transmission loss

Transmission performance of TLF is mainly determined by a core size (to determine the CL) and a wall thickness (to determine the transmission band) while the bending loss is controllable by a hole size. Hence, the characteristics of TLF can be tailored by adjusting the independent design parameters toward bending insensitive low loss design. In the 1TLF design, however, there is a trade off between low bending loss and low transmission loss^19^. As confirmed, a smaller cladding hole size is desired to achieve a low bending loss. However, the small cladding hole effectively reduces a distance between a core and a fiber jacket tube, resulting in a leakage loss increase. The 2TLF structure is not bound to this trade-off. The second cladding layer keeps the distance large enough to avoid the excess leakage loss without sacrificing the capillary hole size, hence promising better bending performance.

**Figure 6:**
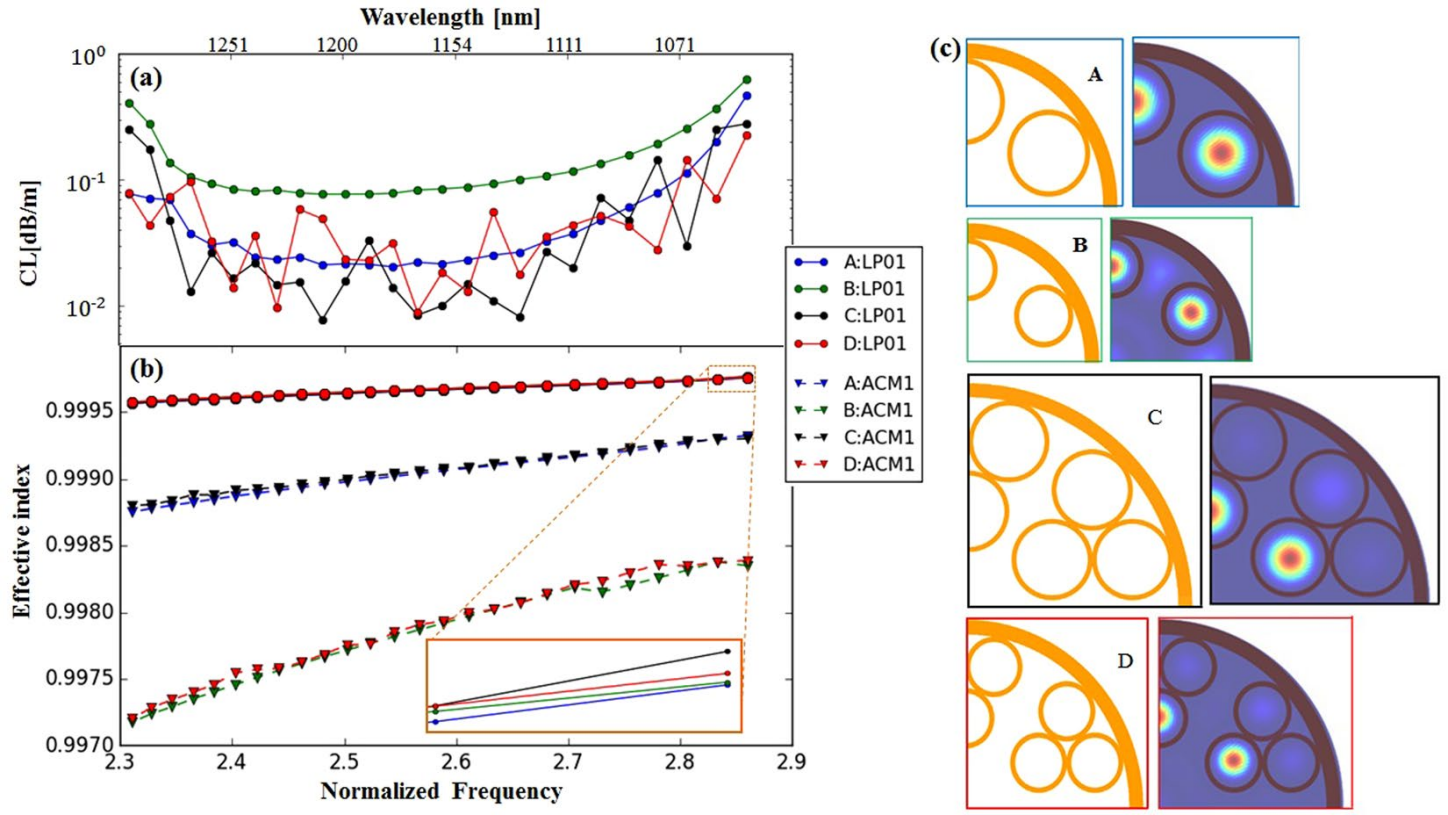
(**a**) Simulated confinement loss of the fundamental mode; (**b**) simulated refractive index of the fundamental mode and airy cladding mode. (**c**) All the structures have the same core size 32.0 *μm* and the same wall thickness 1.43 *μm*. The hole diameter of the cladding capillaries in structure A and C is 20.0 *μm* while that in structure B and D is 13.14 *μm*. The airy cladding mode inside the first cladding ring has the closest effective index to the core modes and is shown in the right side of the figure.

We confirm this additional benefit of 2TLF in Fig. 6. We theoretically compare 4 fiber designs with various hole sizes. The first two structures represent 1TLF, and the others 2TLF. All the structures have the same core diameter 32.0 *μm* and same wall thickness 1.43 *μm*, to make a fair comparison. The only difference between structure A and B, and structure C and D is the capillary hole diameter. The hole diameter in structure A and C is 20.0 *μm* while that in structure B and D is reduced to 13.2 *μm*. Figure 6(b) illustrates effective index of the airy cladding mode in the first cladding ring (ACM1). A beam profile of ACM1 is shown in Fig. 6(c). The index gap between the ACM1 and the core mode can represent the coupling strength, hence a transmission loss. The core mode effective index is nearly same for the all fibers because of the same core diameter. In addition, the effective refractive index of ACM1 in structures A and C are much larger than structures B and D as shown in Fig. 6(b). Consequently, the coupling between core modes and the ACM1 in structure A and C are stronger. This explains why shrinking the cladding capillary hole can reduce the bending loss.

Subsequently, it can be expected that both structure B and D would have lower bending loss than fiber A and C, respectively due to the smaller hole size. However, Fig. 6(a) shows the CL of structure B is much higher than that of structure A. This high CL accounts for close proximity of core to the jacket tube. In 1TLF, the attained low bending loss from the small hole size is compensated by the high CL. Fortunately, 2TLF is free from this constraint as indicated in Fig. 6(a). The second layer provides a sufficient distance between core modes and fiber jacket to prevent the undesired leakage. Hence, 2TLF provides a highly engineerable platform, with freely adjustable individual design parameters of *D*, *t* and *d* without trade-offs. Besides, the bending loss of 2TLF should not be higher than their 1TLF counterpart because adding the second cladding layer does not increase the bending loss^33^.

## Discussion

Contradicting requirements in a TLF design lead to compromises in anti-resonant fiber performances. The key design parameters including core size, wall thickness and capillary hole size are often interlinked, provoking trade-offs in fiber characteristics. Adding a second cladding layer to the TLF can resolve the contradicting requirements and allows individual control of the parameters.

We both theoretically and experimentally present benefits of 2TLF for a small core low loss anti-resonant fiber that are promising for the applications of gas-light interaction and mid-infrared transmission. Besides, bending loss dependence on the capillary hole size was experimentally demonstrated. We also showed the ability of 2TLF to achieve low bending loss without sacrificing the confinement loss by fulfilling small capillary hole size requirement as well as separation of the core and the jacketing tube. Hence, the second cladding layer could potentially bring benefits to advances of anti-resonant fiber technology by allowing independent control of key design parameters to fulfill the design purposes without compromise.

## Methods

### Hollow core fiber fabrication

HSQ 300 tubes (16 *mm* × 20 *mm*) and HSQ 300 rods (12 *mm*) are purchased from Heraeus. These tubes and rods are then drawn into smaller capillaries and rods respectively. The drawn capillaries and rods are then stacked together and jacketed by another HSQ 300 tube to form a preform. Subsequently, the preform is pulled into air core fibers in-house. The structures of the fibers are shown in Fig. 1(a) and (b).

### Background loss measurement

The methodology to determine the background loss of the hollow core fibers is shown in Fig. 1(c). We use a supercontinuum source (SC400 from Fianium) as the light source. The light is coupled into the fiber using a plane-convex lens with 25 *mm* focal length (L1). The fiber is first bent to a circle with 15 *cm* diameter in order to filter out the higher-order modes. The another end of the fiber (point A) is then inserted into a bare fiber adapter and connected to OSA to measure a transmission spectrum. The fiber under test (FUT) is cut to a shorter length (point B) for another transmission spectrum measurement. The deviation between the two transmission spectra reveals an attenuation loss of the FUT. To ensure that we measure the fundamental mode loss, the output light from the point B is directed to a CMOS camera DCC1545M (from Thorlabs) by using a 50x microscope objective. From the camera image, no indication of higher-order modes can be observed, confirming that the fiber bending effectively suppress the higher-order modes.

### Bending loss measurement

Figure 3(a) depicts our schematic to measure the bending loss. The laser beam from a supercontinuum source (SC400 from Fianium) is coupled into the FUT using a plane-convex lens (L1). The FUT is bent to a half circle with diameter *D*_*b*_. After propagating through the bent fiber, the laser beam is couple into a single mode fiber (SMF) by a bare fiber adapter (FA). The SMF is connected to an OSA to measure the transmission spectrum. This method is commonly used to determine bending losses of hollow core fibers^41^. To control a bending direction of the tested fiber, the FUT is mounted on a rotational stage, and the end facet is monitored under a CMOS camera (DCC1545M from Thorlabs) to confirm the bending direction along a cladding gap. When the fiber is revolved on the rotational stage, the bending direction changes accordingly. To ensure the fiber is not twisted, the fiber end is not bounded when rotated.

## Electronic supplementary material

Supplementary Information
