# Supplementary material for: Function of second cladding layer in hollow core tube lattice fibers
Source: Sci Rep. 2017 May 9;7:1618. doi: 10.1038/s41598-017-01839-5 (PMC5431655; doi:10.1038/s41598-017-01839-5)
Supplement: Supplementary file 1 — Supplementary Information [file 41598_2017_1839_MOESM1_ESM.pdf]

Function of a second cladding layer in hollow core tube lattice fibers

Xiaosheng Huang[1] , Seongwoo Yoo[1,*], and KenTye Yong[1]

[1]The Photonics Institute, School of Electrical and Electronics Engineering, Nanyang Technological University, Singapore 639798, Singapore
*seon.yoo@ntu.edu.sg

Supplementary Figure S1 compares the simulated confinement loss (CL) of a tube lattice fiber (TLF, see the inset in Supplementary Fig. S1) obtained by Polymode (an open source software based on the wave vector expansion method) to the published results in [1], by Comsol. The discrepancy between both is within 10%, which indicates our results obtained with Polymode are reliable to study the property of TLF.

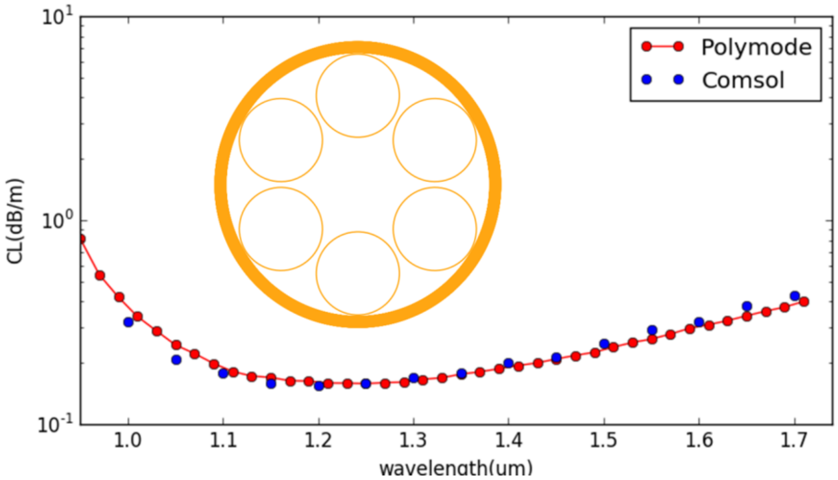

Supplementary Figure S1. Confinement loss calculated by Polymode (used in this study) and Comsol in [1]. Inset: Fiber structure for the comparison, with a core radius R = 15 μm and wall thickness t = 0.42 μm [1]. The Red line shows the confinement loss by Polymode while blue dots are cited from [1].

The method to determine a fiber bending direction is illustrated in Supplementary Fig. S2. The side of a fiber end is marked with a red line, which is aligned to one of the split cladding compartments. Subsequently, the fiber is bent perpendicular to the marked line, which is toward one of the cladding splits (as indicated by the red arrow). Under a microscope, we can identify the corresponding split gap that the fiber is bent toward.

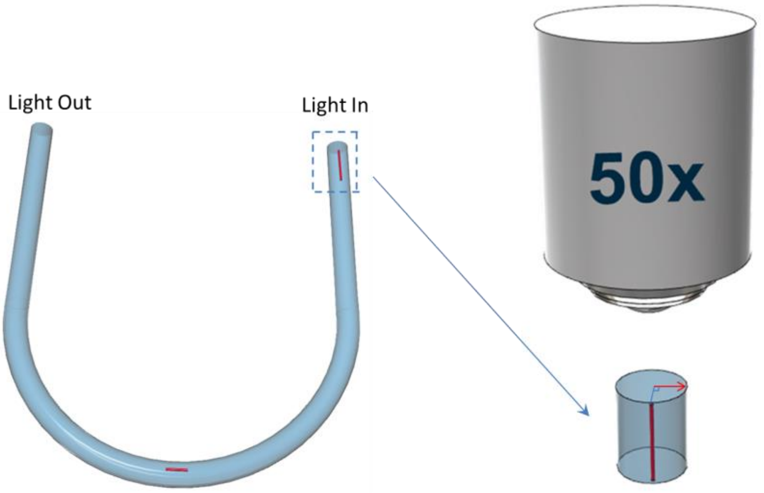

Supplementary Figure S2. Shows the schematic to determine the bending direction of the fiber. The red arrow on the input fiber end indicates the bending direction.

When a preform is pulled into a hollow core fiber, differential pressure is applied between the air core and the capillary holes to maintain the structure uniformity. As illustrated in Supplementary Fig. S3, an end of the preform is connected to one gas channel (Gas Channel 1) to build positive pressure in the capillaries. The core area is blocked to prevent influx gas flow (grey area). Another gas channel is used to control gas pressure in the core via the side hole as indicted by Gas Channel 2 in the figure. By adjusting the gas flows between the core and the cladding areas, the required differential pressure is attainable.

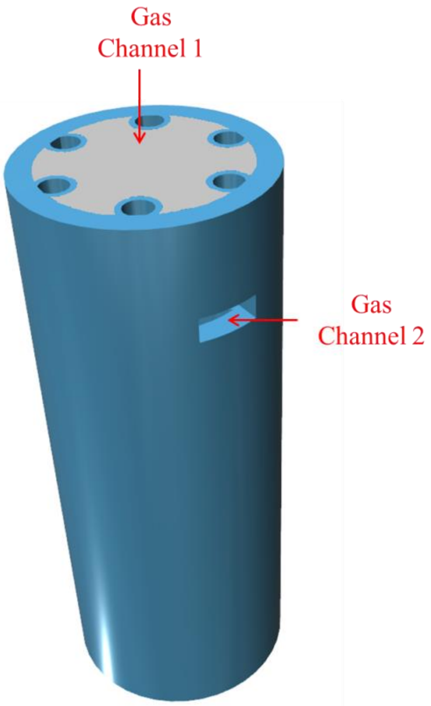

Supplementary Figure S3. Shows the schematic to control the gas pressure difference between core and cladding areas during a fiber drawing process.

The fabricated fibers contain layers of solid rods placed between the fiber jacket tube and the cladding capillaries as illustrated in Supplementary Fig. S4 (Structure A). The structure can be simplified to structure D, assuming the rods as a part of the

jacket tube wall. Here, we would like to justify this assumption. Supplementary Figure S4 presents a calculated confinement loss (CL) from structure A to structure D, with progressive simplification of the cladding structure. As shown, the performance of structures A, B and C are nearly the same. The structure D marks a bit lower CL because of a reduced contacting area between the jacketing tube and the capillaries as compared to structure C. The reduced contact area helps to suppress the CL. We further confirm this with the structure E where the touching area becomes even larger. As expected, the CL of structure E is the highest while structure D offers the lowest CL thanks to the minimum touching area. As the CL discrepancy between structure A and D is marginal (within 25%), we decide to use simplified structure D to facilitate the simulation.

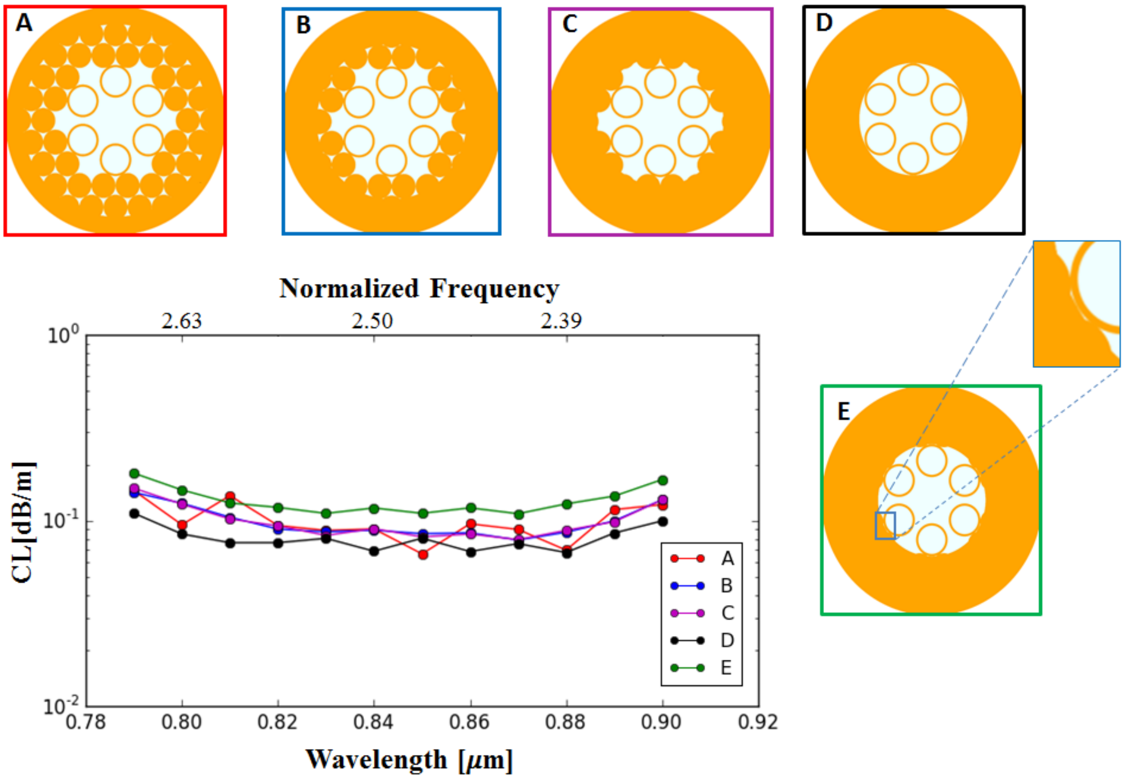

Supplementary Figure S4. Compares the CL of different structures (A to E) within the third transmission band (2<*F*<3, normalized frequency *F* is defined in the manuscript). All the structures have the same core diameter (10.0 μm), capillary size (14.3 μm) and wall thickness (1.0 μm).

References

[1] F. Poletti, “Nested antiresonant nodeless hollow core fiber,” *Optics express*, vol. 22, no. 20, pp. 23807–23828, 2014.
